# Diarylheptanoids/sorafenib as a potential anticancer combination against hepatocellular carcinoma: the p53/MMP9 axis of action

**DOI:** 10.1007/s00210-023-02470-0

**Published:** 2023-05-05

**Authors:** Alaa Elmetwalli, Thoria Diab, Aisha Nawaf Albalawi, Sabry Ali El-Naggar, Ali H. El‑Far, Amira Radwan Ghedan, Eman Saad Alamri, Afrah Fatthi Salama

**Affiliations:** 1 Department of Clinical Trial Research Unit and Drug Discovery, Egyptian Liver Research Institute and Hospital (ELRIAH), Mansoura, Egypt; 2 Division of Biochemistry, Department of Chemistry, Faculty of Science, Tanta University, Tanta, Egypt; 3 Department of Biology, University of Haql College, University of Tabuk, Tabuk, 71491 Saudi Arabia; 4 Zoology Department, Faculty of Science, Tanta University, Tanta, Egypt; 5 Department of Biochemistry, Faculty of Veterinary Medicine, Damanhour University, Damanhour, 22511 Egypt; 6 Nutrition and Food Science Department, University of Tabuk, Tabuk, 71491 Saudi Arabia

**Keywords:** Diarylheptanoid, Antioxidant, Cancer-targeting, HCC

## Abstract

Hepatocellular carcinoma (HCC) is a serious and potentially fatal form of cancer associated with liver damage. New anticancer drugs are increasingly needed due to the increasing number of cancer cases every year. In this study, diarylheptanoids (DAH) from *Alpinia officinarum* were examined for their antitumor activity against DAB-induced HCC in mice, as well as their ability to reduce liver damage. Assays for cytotoxicity were conducted using MTT. The DAB-induced HCC Swiss albino male mice were given DAH and sorafenib (SOR) either as single treatments or in combination, and the effects on tumour development and progression were monitored. Malondialdehyde (MDA) and total superoxide dismutase (T-SOD) were evaluated along with biomarkers of liver enzymes (AST, ALT, and GGT). The apoptosis-related gene (*CASP8*), the apoptosis-related gene (*p53*), the anti-inflammatory genes (*IL-6*), the migration-related gene matrix metalloprotease-9 (*MMP9*), and the angiogenesis-related gene vascular endothelial growth factor (*VEGF*) were assessed using qRT-PCR in the hepatic tissue. As a final step, DAH and SOR were docked with *CASP8* and *MMP9* via molecular docking to propose potential mechanisms of action. Our results revealed that the combination of DAH and SOR has a potent inhibitory effect on the growth and viability of the HepG2 cell line. The outcomes demonstrated that DAH and SOR-treated HCC-bearing mice displayed a reduction in the tumour burden and liver damage as demonstrated by (1) parameters of repaired liver function; (2) low levels of hepatic MDA; (3) elevated levels of hepatic T-SOD; (4) *p53*, *IL-6*, *CASP8*, *MMP9*, and *VEGF* downregulation; and (5) enhanced hepatic structure. The best results were revealed in mice that were co-treated with DAH (given orally) and SOR (given intraperitoneally). The docking study also proposed that both DAH and SOR could inhibit *CASP8* and *MMP9*’s oncogenic activities and had a high affinity for these enzymes. In conclusion, according to study findings, DAH enhances SOR antiproliferative and cytotoxic effects and identifies their molecular targets. Furthermore, the results revealed that DAH was able to boost the anticancer effects of the drug SOR and reduce liver damage caused by HCC in mice. This suggests that DAH could be a potential therapeutic agent against liver cancer.

## Introduction

As cancer cells divide without the usual growth signals, they are unable to respond to the normal cell cycle checkpoints that would normally slow their growth. This uncontrolled cell division leads to the formation of a tumour, which can then invade nearby organs or spread to distant organs by metastasis (Mahmud et al. 2022). Currently, the only approved treatments are limited to those who have early-stage HCC, and even these treatments are not very effective in the long term. In addition, many of the existing treatments are associated with severe side effects, making them not a viable option for those with advanced-stage HCC (Kelley 2022). Therefore, new and more effective treatments are desperately needed to improve the prognosis and quality of life for those with HCC. Besides surgery, complementary and alternative medicine is viewed as a viable option for increasing the effectiveness of anticancer medications and reducing their detrimental side effects (Gezici and Şekeroğlu 2019).

There is still a lot of skepticism from the scientific community regarding the effectiveness of herbal treatments. In addition, there is a lack of clinical trials to back up their efficacy. However, due to their low cost and accessibility, herbal treatments are still widely used by health practitioners looking for more natural methods of treatment (Ozioma and Chinwe 2019). In recent years, phytochemicals and secondary metabolites of natural products have been found to have anti-inflammatory and antioxidant properties, which means that they can help to reduce inflammation and protect cells from damage caused by free radicals. This can help to reduce the risk of cancer and other chronic diseases (Al-Dabbagh et al. 2019).

Specifically, there are many medicinal plants used in traditional medicine that contain significant amounts of natural antioxidants. It has been reported that these plants can protect against cancer, atherosclerosis, cerebral cardiovascular events, diabetes, hypertension, and Alzheimer’s (Akbari et al. 2022). Additionally, medicinal plants offer an array of other health benefits, such as improved digestive health and immune system enhancement. This is due to the fact that they contain a variety of antioxidants, polyphenols, and other compounds that have been shown to have protective effects against a range of diseases (El-Dakhly et al. 2020) (Hamza et al. 2020).

The antioxidants found in natural products are extremely effective at neutralizing reactive oxygen species (ROS) that can lead to oxidation. Natural antioxidants such as flavonoids, tannins, coumarins, curcuminoids, xanthones, phenolics, and terpenoids are responsible for the antioxidant and pharmacological properties of medicinal plants (Hamza et al. 2023).

Plant extracts (tea and grapeseed) and herbal products (rosemary, thyme, oregano), as well as spices such as pepper and clove, were used in folk medicine that plays a significant role in primary health care (Ozdal et al. 2021). These plant-based ingredients and products can be used to not only flavour food (Sieniawska et al. 2020) but also to treat a wide variety of medical conditions, providing support for the age-old practice of folk medicine (Qadir and Raja 2021).

Several pharmacological properties of *Alpinia officinarum* have been reported, including anti-inflammatory, antioxidant, antiemetic, antibacterial, and cytotoxic effects (Ding et al. 2019). In a recent study, bioactive compounds derived from *A. officinarum*’s rhizomes were also shown to have an anti-inflammatory effect on lipopolysaccharide-stimulated HepG2 cells. Tumour necrosis factor-*α (TNF-α)*, interleukin-6 (*IL-6*), interleukin-1*β (IL-1β)*, and pro-inflammatory cytokine gene expression were reduced in a dose-dependent manner in response to the chemical constituents of DAH, implying that they could be used to treat inflammatory diseases (Elgazar et al. 2018). Sorafenib (SOR) is an oral tyrosine kinase inhibitor that plays on the RAF/MEK/ERK pathway, the VEGFR, and PDGFR-tyrosine kinases ((Ma et al. 2021; Bhat et al. 2022). DAH and SOR are still inconclusive combinations and further studies are required to determine the full effects of this combination therapy. Therefore, we hypothesized that DAH and/or sorafenib can inhibit the growth of HCC cells by suppressing the expression of oncogenic proteins and upregulating the expression of tumour suppressor proteins. We conducted detailed in *vitro* and *in vivo* studies to validate this hypothesis.

## Materials and methods

### Cell culture

DMEM (BioWhittaker^®^, USA) was used to culture HepG2 (liver cancer cell line) and WI-38 normal cell lines. They grew in Dulbecco’s modified Eagle’s medium (Nuair, Germany) supplemented with 10% foetal bovine serum (Sigma, USA) at 37 °C with 5% carbon dioxide, in a humidified incubator with 100 g/ml penicillin and 100 g/ml streptomycin, and supplemented with 10% foetal bovine serum (Sigma, USA). In this study, three independent experiments were conducted.

### Cell viability assay

MTT colourimetric assay kit (Sigma-Aldrich, USA) was used to figure out the viability of HepG2 and WI-38 cells after treatment with DAH and SOR. For 24 and 48 h, cells were seeded in 96-well plates at 37 °C, and cultured overnight before being treated with DAH and SOR at concentrations of 1.56–100 µg/ml. For 4 h, each well was incubated at 37 °C in the dark with MTT working solution (100 ml). In a 5-min procedure, 100 ml of DMSO was added to each well and the purple formazan crystals were dissolved. A microplate reader (BIO-RAD PR4100, USA) was used to measure optical density (OD) at 570 nm. For the calculation of 50% inhibitory concentration (IC_50_), the viability of treated cells was compared with control untreated cells, which were considered to have 100% viability (Chung et al. 2015).

The IC_50_ value is used to measure the efficacy of a drug in inhibiting a particular cell line (HepG2). By combining DAH with SOR treatment, the cells were exposed to different concentrations of the drug. After 48 h of incubation, the MTT assay was performed to measure the number of viable cells and these data were analysed to determine the IC_50_ value. Practically, IC_50_ values were determined using a combination of DAH with SOR treatment on HepG2, which was treated with (IC_10_–IC_50_) doses of DAH along with SOR (1.0–40.0), and was then incubated for 48 h before performing the MTT assay (Bahuguna et al. 2017) as described above.

### Statement of ethics

The approval was based on the guidelines set forth by Tanta University’s Faculty of Science (IACUC) for animal care and use. These guidelines include the treatment of animals, proper housing, and the use of procedures that minimize the potential for pain and distress. The protocol’s approval number is (IACUC-SCI-TU-0239). The ARRIVE guidelines were strictly followed during all procedures to ensure that animals used in research are given the most suitable possible care and that all procedures are conducted humanely and ethically. The experiment was conducted on male Swiss albino mice weighing 20–25 g and aged 10–12 weeks. During the course of the experiment, the mice were kept in a controlled environment. As part of the experiment, the mice were kept in an environment with a 24-h cycle, where the temperature was controlled. As far as the animals were concerned, they were fed a typical diet and provided with water ad libitum, as well.

### Preparation of enriched DAH fraction from rhizomes of *Alpinia officinarum*

With an electric grinder, we ground the rhizomes and extracted them with 80% acetone to give 250 g of total extract in 1 l of distilled water. We then shook them with ethyl acetate until they turned colourless. Using a vacuum, 120 g of the ethyl acetate fraction was collected and evaporated from the organic layer.

### Experimental design

A total of 96 male Swiss albino mice were assigned to 8 groups (*n* = 12). The groups were as follows:

1. Group 1 (control): normal mice orally administrated with distilled water and a normal low-protein diet for three months.
2. Group 2 (Gp2): normal mice administrated DAH alone (orally by fine pipette at a dose of 100 mg/kg body weight of mice daily) dissolved in a carboxymethylcellulose (CMC) 0.5%.
3. Group 3 (Gp3): normal mice were injected with SOR alone (i.p., at a dose of 15 mg/kg daily).
4. Group 4 (Gp4): normal mice were injected with DAH and SOR with doses revealed in groups 2 and 3 respectively.
5. Group 5 (Gp5): mice were induced for HCC by p-dimethylaminoazobenzene (DAB) as an initiator (orally at a daily dose of 165 mg/kg per mouse) dissolved in alcohol 70% and an aqueous solution of phenobarbital (PB) as a promoter (orally by fine pipette at dose 0.06 ml of 0.05% concentration per mouse) mixed with the basal diet for 60 days.
6. Group 6 (Gp6): mice were induced for HCC as in group 5 and treated with SOR alone (i.p. at a dose of 15 mg/kg daily for a month).
7. Group 7 (Gp7): mice were induced for HCC as in group 5 and treated with DAH alone (at the dose of 100 mg/kg body weight by oral gavage).
8. Group 8 (Gp8): mice were induced for HCC as in group 5 and treated with SOR and DAH at doses as revealed in groups 6 and 7, respectively.

### Sampling

Mice that were overnight fasting for 24 h were sacrificed through isoflurane at the end of the trial period. Blood samples were centrifuged for 15 min at 3000 × g to get clear, non-hemolysed sera after the final treatment. The sera were placed in Eppendorf tubes with labels that were immediately moved to −20 °C to be frozen for biochemical analysis.

As soon as the livers were taken after euthanasia, some were preserved in 10% formalin for pathological examination, while others were homogenized for biochemical analysis or frozen at −70 °C (RNA extraction).

### Assessment of biochemical parameters

Alanine transaminase (ALT), aspartate transaminase (AST), gamma-glutamyltransferase (GGT), and alpha-fetoprotein (AFP) were measured using commercially available kits to evaluate the blood level of liver damage (Biomed Diagnostics, Cairo, Egypt). Colourimetric measurement of malondialdehyde (MDA) and total superoxide dismutase (T-SOD) was conducted in the hepatic tissue using kits obtained from Biomed Diagnostics (Cairo, Egypt), as previously mentioned by Attia et al. (2022).

### Real-time PCR

Caspase-8 (*CASP8*), *IL-6*, *p53*, matrix metalloproteinase-9 (*MMP9*), and vascular endothelial growth factor (*VEGF*) mRNA expression variations in liver tissues from all groups were assessed using real-time PCR. After being extracted, total RNA was reverse-transcribed into cDNA using kits obtained from Thermo Scientific, USA. The primer sequences were as follows: F: 5′ AGAGTCTGTGCCCAAATCAAC 3′ and R: 5′ GCTGCTTCTCTCTTTGCTGAA 3′ for *CASP8*; F: 5′ GGTACATCCTCGACGGCATCT 3′ and R: 5′ GTGCCTCTTTGCTGCTTTCAC 3′ for *IL-6*; F: 5′ CGGGAAGACAATAACTGCACCC 3′ and R: 5′ CGGTTAGCAGTATGTTGTCCAGC 3′ for *p53*; F: 5′ GATGCGTGGAGAGTCGAAAT 3′ and R: 5′ CACCAAACTGGATGACGATG 3′ for *MMP9*; F: 5′ CATCTTCCAGGAGTACCCCGA 3′ and R: 5′ CACTCCAGGGCTTCATCGTT 3′ for *VEGF*. Conditions for the thermal and melting curves were performed as heretofore designated (Attia et al. 2022). Using the 2^−∆∆Ct^ technique, the fold change in gene expression was calculated.

### Histopathological investigation

For histological analysis, liver tissues were cleaned in xylene, fixed in paraffin wax, and segmented at a thickness of 5 mm, using ethanol concentrations as a desiccant.

### Molecular docking

The protein sequence of mouse *CASP8* and human *MMP9* is present as predicted sequences. Therefore, we perform molecular docking of DAH and SOR against human *CASP8* (target site PDB ID: 1I4E) and human *MMP9* (target site PDB ID: 1GKC), because human and mice *CASP8* and *MMP9* have the same binding sites for DAH and SOR. We first downloaded it from the RCSB PDB database (https://www.rcsb.org/) and prepared it with BIOVIA Discovery Studio Visualizer software (https://discover.3ds.com/discovery-studio-visualizer-download). In addition, the 3D structures of DAH and SOR were obtained from the PubChem database (https://pubchem.ncbi.nlm.nih.gov/). The binding free energy, binding affinity (p*Ki*), and the ligand efficiency of DAH and SOR against human *CASP8* (1I4E) and *MMP9* (1GKC) were determined using InstaDock software. Finally, BIOVIA Discovery Studio Visualizer software did the visualization of target-ligand interaction.

The determination of the possible binding potentials of DAH to molecular docking of DAH and the promotor sequences of *Mus musculus CASP8*, *IL-6*, *p53*, *MMP9*, and *VEGF* genes were done to figure out the inhibitory effect of DAH on the gene level. The promotor sequences were retrieved from the EPD database (https://epd.epfl.ch/). The docking between receptors and ligands was done through PatchDock Server (https://bioinfo3d.cs.tau.ac.il/PatchDock/) and the data were visualized using BIOVIA Discovery Studio Visualizer software.

### Statistical analysis

The data were studied using GraphPad Prism 9.0. The outcomes of the experiment were presented as mean ± SD. IC_50_ values were calculated based on a sigmoid nonlinear regression (Lyles et al. 2008). One-way analysis of variance (ANOVA) and post *hoc* Tukey test for multiple comparisons was used to analyse the data. Statistics were deemed to be significant if the value had a *P* value of < 0.05.

## Results

### DAH enhances SOR cytotoxicity in liver cancer cell line

**Fig. 1:**
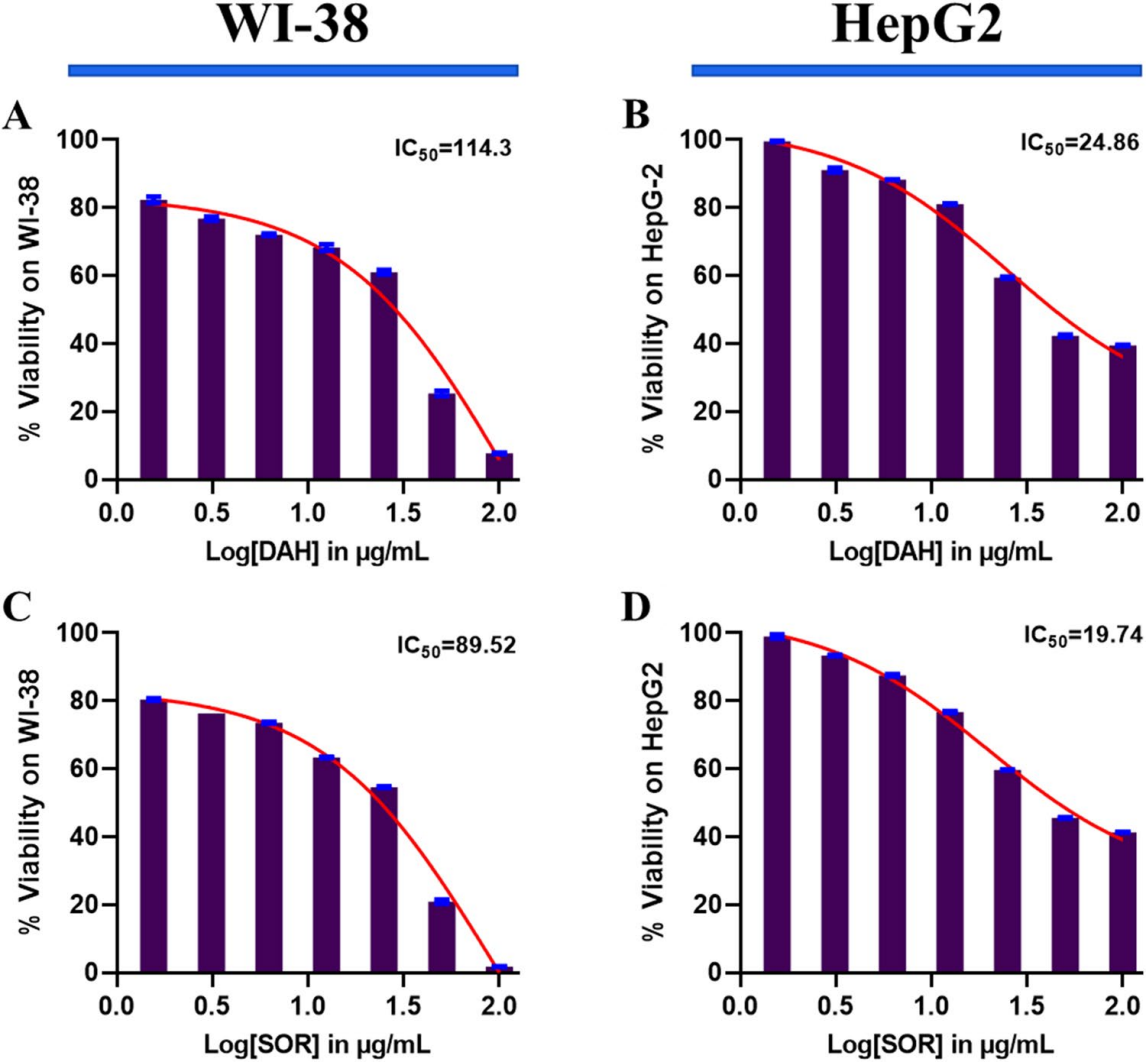
Effect of DAH and SOR on cell viability of WI-38 and HepG2 cells. Different concentrations of DAH (**A** and **B**) and SOR (**C** and **D**) were applied to cells for 48 h before MTT assay was conducted. All experiments were repeated three times and the results were expressed as cell viability (% of control)

In WI-38 and HepG2 cells treated for 48 h, DAH and SOR were found to have antiproliferative effects on the cells. SOR and DAH IC_50_ values were determined using dose-response curves. SOR- and DAH-treated cells inhibited cell proliferation dose-dependently compared to control cells. According to HepG2 cells, the IC_50_ for DAH and SOR was 24.86 µg/ml and 19.74 µg/ml, respectively. WI-38 cells, however, exhibited IC_50_ values of 114.3 and 89.52 µg/ml, respectively (Fig. 1A–D). These results suggested that DAH and SOR are effective at inhibiting cell proliferation at different concentrations, with DAH being more potent than SOR in HepG2 cell lines. Furthermore, it appeared that the WI-38 cells are more resistant to the effects of DAH and SOR than the HepG2 cells. The IC_50_ values for the combination treatments were examined and were revealed in Table 1. The IC_50_ value for SOR was significantly reduced with variable concentrations of DAH (IC_10_–IC_50_) in HepG2 cells, which were combined with the IC_50_ value for SOR. The maximum reduction in IC_50_ (10.79 ± 1.02) was detected when DAH’s predetermined IC_50_ was combined with a variable concentration of DAH. The results of the combination treatments demonstrated that the IC_50_ value for SOR was significantly reduced when administered with variable concentrations of DAH. This indicates that the combination of SOR and DAH is more effective than either drug alone at inhibiting cell growth.

**Table 1:** % change for SOR IC_50_ in combination with different doses of DAH against HepG2 cancer cell line

	IC_50_ of SOR (% change)
	HepG2
Sorafenib (SOR) alone	**28.56 ± 2.35**
DAH + SOR IC_10_	27.14 ± 1.56^ns^
DAH + SOR IC_20_	21.19 ± 1.14^*^
DAH + SOR IC_30_	18.21 ± 0.89^**^
DAH + SOR IC_40_	13.25 ± 0.78^**^
DAH + SOR IC_50_	10.79 ± 1.02^***^

Each experiment was expressed as a mean ± SD of three independent experiments. A student paired *t*-test was used to figure out if the IC_50_ values (µg/ml) for sorafenib and the IC_50_ values for the corresponding drugs (DAH) were significantly different (*ns*, non-significant, * *p* ˂ 0.05, ** *p* ˂ 0.01, and *** *p* ˂ 0.001)

### Influence of DAH and/or SOR on liver functions

**Fig. 2:**
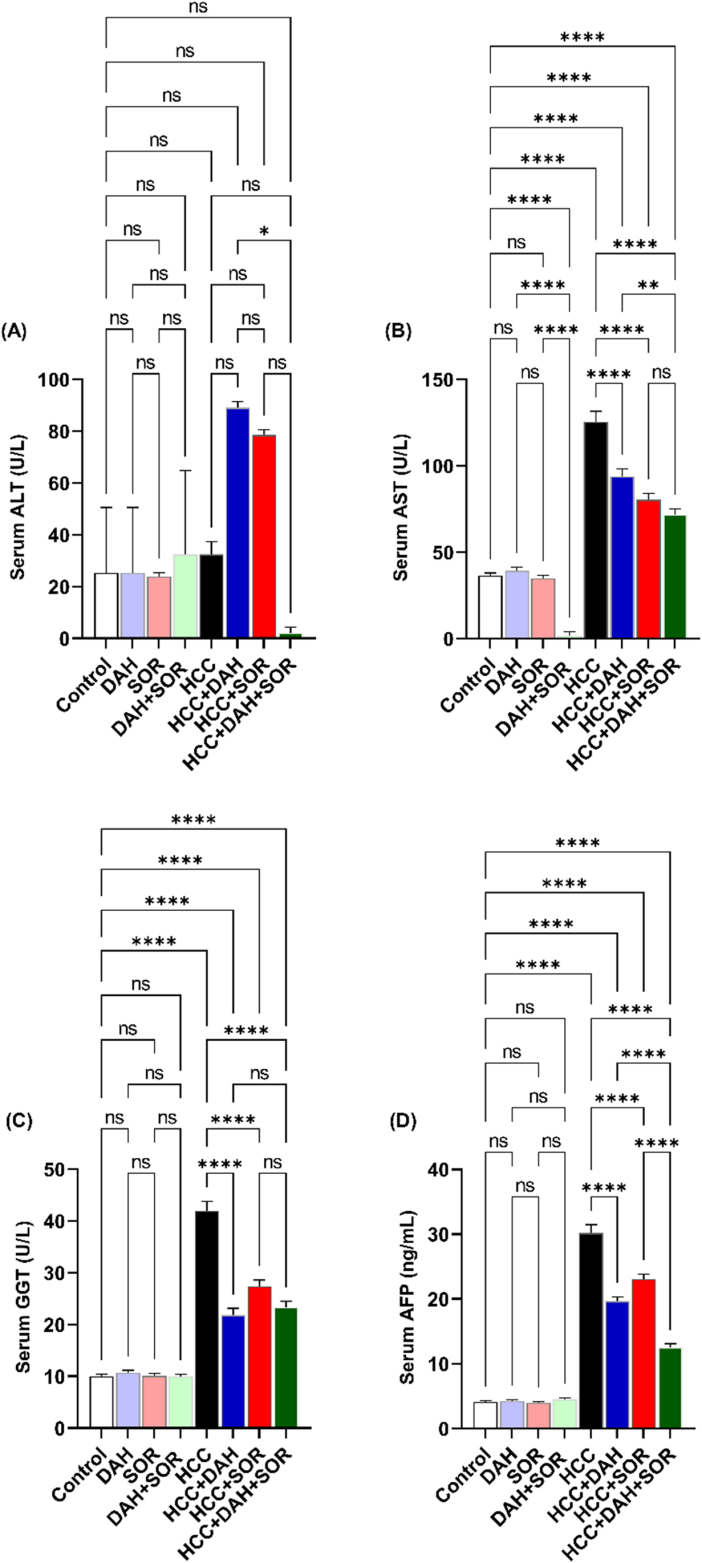
Serum markers. **A** Serum alanine aminotransferase (ALT). **B** Serum aspartate aminotransferase (AST). **C** Serum gamma-glutamyl transferase (GGT). **D** Serum alpha-fetoprotein (AFP). Data were expressed as the mean ± SD; * (*p* ˂0.05), ** (*p* ˂ 0.01), and **** (*p* ˂ 0.0001)

DAH and/or SOR-treated HCC mice exhibited significantly decreased ALT and AST blood levels when compared to control HCC mice. These serum biochemical markers significantly improved in the HCC + SOR, HCC + DAH, and HCC + DAH + SOR groups. This is likely due to the fact that the treatments can target and eliminate tumour cells, which in turn causes liver enzymes to decrease. The combination treatment of DAH and SOR is even more effective at reducing ALT and AST levels, suggesting that the two treatments worked synergistically to reduce the tumour burden in the liver. Compared to HCC mice that were not treated, DAH and/or SOR-treated HCC mice displayed considerably lower blood levels of GGT. HCC + DAH and HCC + DAH + SOR demonstrated the highest improvement in these serum biochemical markers, followed by HCC + SOR. Similarly, the serum ALT, AST, and GGT levels in the HCC + DAH, HCC + SOR, and HCC +DAH +SOR groups were all significantly higher than those in the control group (Fig. 2A–C).

### Influence of DAH and/or SOR on liver tumour marker AFP

The liver AFP levels of HCC mice were significantly higher than those of all control groups. As compared to HCC untreated, this level was significantly reduced following SOR and DAH treatment, either alone or combined, with the highest level in HCC + DAH, HCC + SOR, and the lowest level in HCC + SOR + DAH as revealed in Fig. 2D.

### Influence of DAH and/or SOR on oxidative and antioxidative markers

**Fig. 3:**
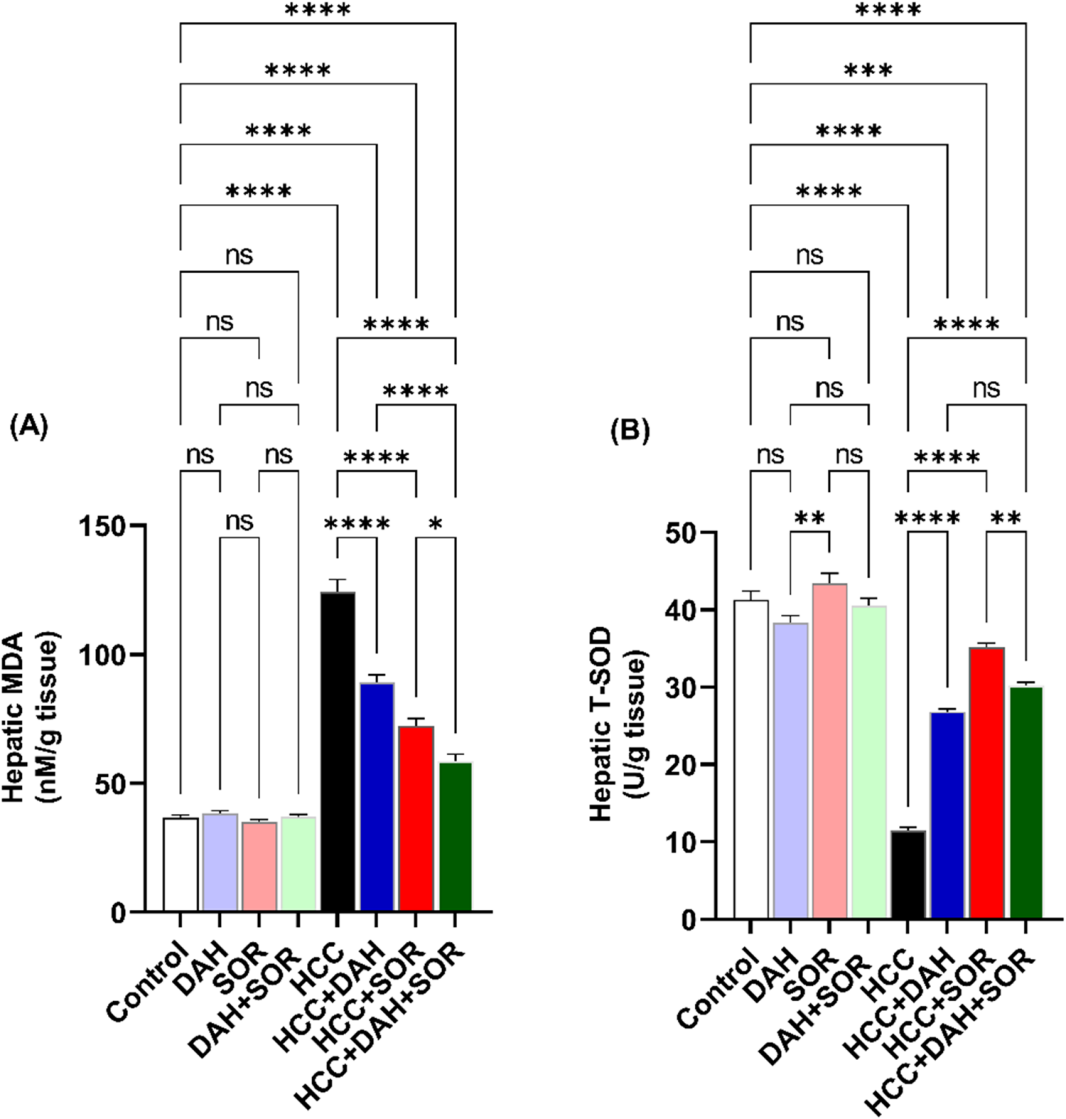
Hepatic oxidative stress and antioxidant markers. **A** Hepatic malondialdehyde (MDA). **B** Hepatic total superoxide dismutase (T-SOD). Data are expressed as the mean ± SD; * (*p* ˂0.05), ** (*p* ˂ 0.01), *** (*p* ˂ 0.001), and **** (*p* ˂ 0.0001)

The liver levels of MDA, an indicator of lipid peroxidation, and T-SOD, an indicator of antioxidant activity, were significantly lower in nontreated HCC-bearing mice than in control mice. We found that three groups—HCC + SOR, HCC + DAH + SOR, and HCC + SOR—had the highest enhancement (lowest MDA and maximum T-SOD). In addition, DAH and/or SOR treatment returned these indicators to levels similar to those of the control groups (Fig. 3A–B). This indicated that the co-administration of DAH and SOR greatly improved the liver’s antioxidant activity and reduced lipid peroxidation in HCC-bearing mice.

### Effect of DAH and/or SOR on the expression of CASP8, IL-6, p53, MMP9, and VEGF mRNAs

**Fig. 4:**
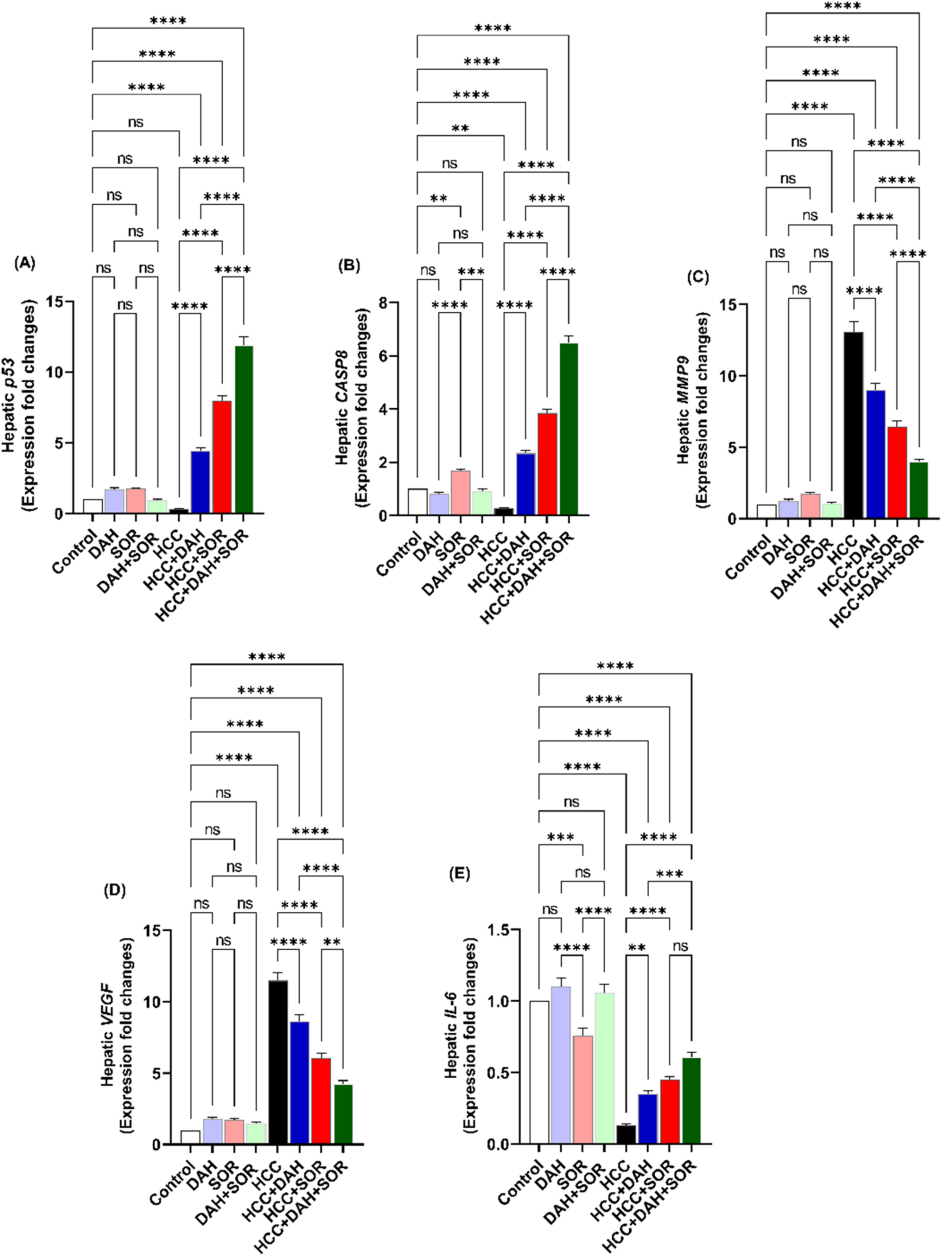
Hepatic mRNA expression fold changes. **A** Hepatic *p53*. **B** Hepatic caspase 8 (*CASP8*). **C** Hepatic matrix metalloproteinase-9 (*MMP9*). **D** Hepatic vascular endothelial growth factor (*VEGF*). **(E)** Hepatic interleukin 6 (*IL-6*). Data were expressed as the mean ± SD; ** (*p* ˂ 0.01), *** (*p* ˂ 0.001) and **** (*p* ˂ 0.0001)

In comparison to the control groups, the HCC group’s hepatic expression of *CASP8* and *p53* was dramatically downregulated, whereas that of *MMP9*, *IL-6*, and *VEGF* was significantly upregulated, according to the qPCR data (Fig. 4). The best enhancement (the highest *CASP8*, *p53*, and the lowest *MMP9*, *IL-6*, and *VEGF*) was shown in HCC + DAH, followed by HCC + SOR, and then the HCC + DAH + SOR group. DAH and/or SOR refurbished the expression of these genes to levels equivalent to the control groups (Fig. 4A–E).

### Histopathological examination

**Fig. 5:**
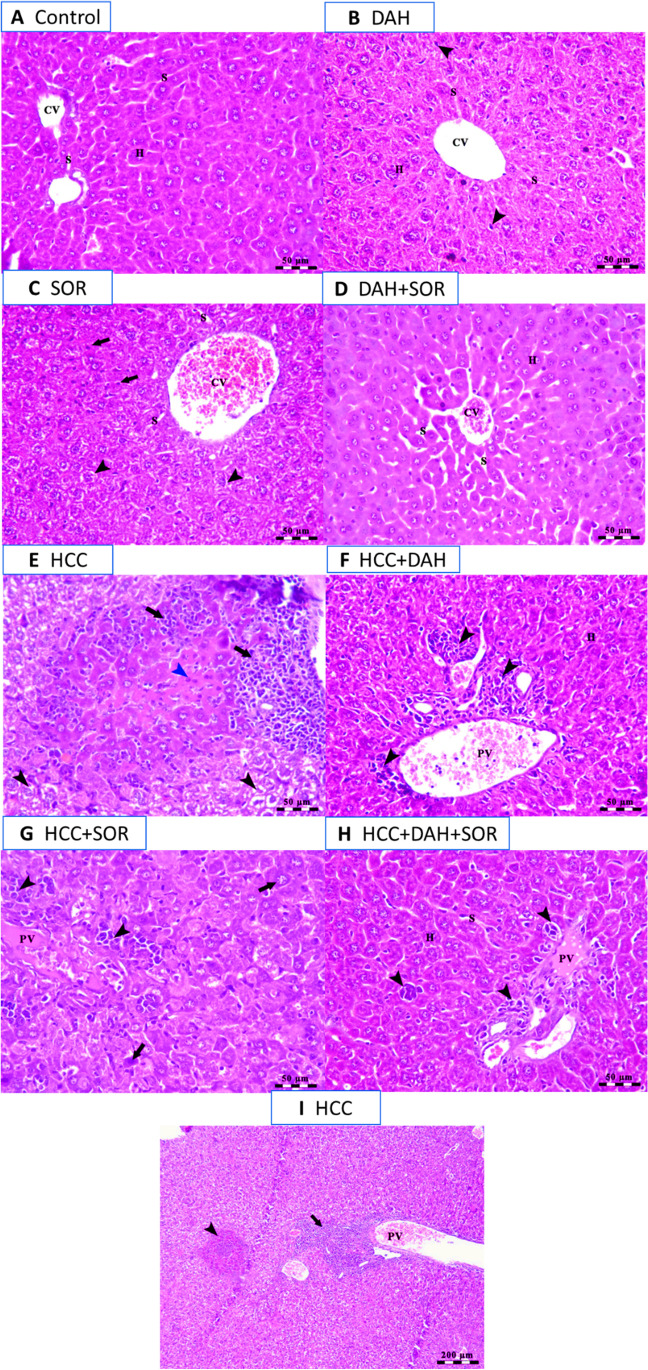
Histopathology assessment. **A** Photomicrograph of the liver of control group showing a normal architecture of hepatic parenchyma; hepatocytes (H) arranged in cord-like pattern separated by blood sinusoids (S) radiating from an intact central vein (CV). **B** Photomicrograph of the liver of DAH-treated group showing polyhedral-shaped hepatocytes (H) arranged in cord-like pattern separated by blood sinusoids (S) radiating from intact CV and presence of Kupffer cells activity (arrowheads). **C** Photomicrograph of centro-lobular area of the liver of SOR-treated group showing cords of hepatocytes separated by blood sinusoids (S) radiating from dilated and congested CV, microsteatosis (arrowheads) and nuclear pyknosis (arrows) of some hepatocytes. **D** Photomicrograph of centro-lobular area of the liver of DAH + SOR-treated group showing mild congestion of CV, intact hepatocytes arranged in cords (H), and mildly dilated blood sinusoids (S). **E** Photomicrograph of the liver of HCC-induced treated group showing loss of hepatic architecture with the presence of a focal area of hepatocellular degeneration with pyknotic nuclei and acidophilic cytoplasm (blue arrowhead) besides vacuolar degeneration of hepatocytes (clear cells; black arrowheads) surrounded by inflammatory cells (arrows). **F** Photomicrograph of the liver of HCC + DAH-treated group showing congestion of portal vein (PV) with the presence of perivascular lymphoid elements (arrowheads) beside intact hepatocytes (H). **G** Photomicrograph of the liver of HCC + SOR-treated group showing congestion of portal vein (PV), ballooning of hepatocytes with nuclear pyknosis (arrows), and presence of diffuse aggregations of lymphoid elements (arrowheads). **H** Photomicrograph of the liver of HCC+ DAH + SOR-treated group showing congestion of portal vein (PV), intact hepatocytes (H) separated by blood sinusoids (S), and presence of small aggregations of lymphoid elements (arrowheads). **I** Photomicrograph of the liver of HCC-induced treated group showing the presence of lymphoid stroma (arrow), focal area of necrosis surrounded by hepatocytes of acidophilic cytoplasm and hyperchromatic nuclei (arrowhead), and congested portal vein (PV). Scale bar = 50 µm

A typical hepatic parenchyma of the control group is exhibited in Fig. 5A, with cord-like hepatocytes and blood sinusoids extending from an intact central vein (CV). Furthermore, the livers of the DAH-treated group contained polyhedral hepatocytes, separated by blood sinusoids originating from intact CV and Kupffer cells (arrowheads) (Fig. 5B). SOR-affected livers disclosed microsteatosis (arrowheads) of certain hepatocytes in the centrilobular region (Fig. 5C) resulting from dilated CV. Furthermore, the DAH + SOR group exhibited mildly congested CV (Fig. 5D), as well as moderately dilated blood sinusoids. There was localized hepatocellular degeneration with pyknotic nuclei and acidophilic cytoplasm within the liver of those treated with HCC (Fig. 5E), along with inflammatory cells within the liver. It is evident in Fig. 5F that the HCC + DAH-treated group has a congested portal vein (PV) and perivascular lymphoid components (arrowheads). There is an aggregation of diffuse lymphoid elements and ballooning hepatocytes, as well as nuclear pyknosis in the HCC + SOR group as revealed in Fig. 5G. Similarly, Fig. 5H of the HCC + DAH + SOR group illustrates intact hepatocytes, along with small lymphoid aggregates (arrowheads), and PV congestion. Figure 5I shows hyperchromatic nuclei and acidophilic cytoplasm in hepatocytes from the HCC treatment group surrounding a necrosis area. From the previous histological examination, our data suggested that the HCC + SOR treatment group has more severe tissue damage compared to the HCC + DAH + SOR group, as evidenced by the presence of lymphoid aggregates, nuclear pyknosis, and portal vein congestion. The HCC treatment group also exhibits the most severe tissue damage, as indicated by the presence of necrotic areas and hyperchromatic nuclei.

### Molecular docking

**Fig. 6:**
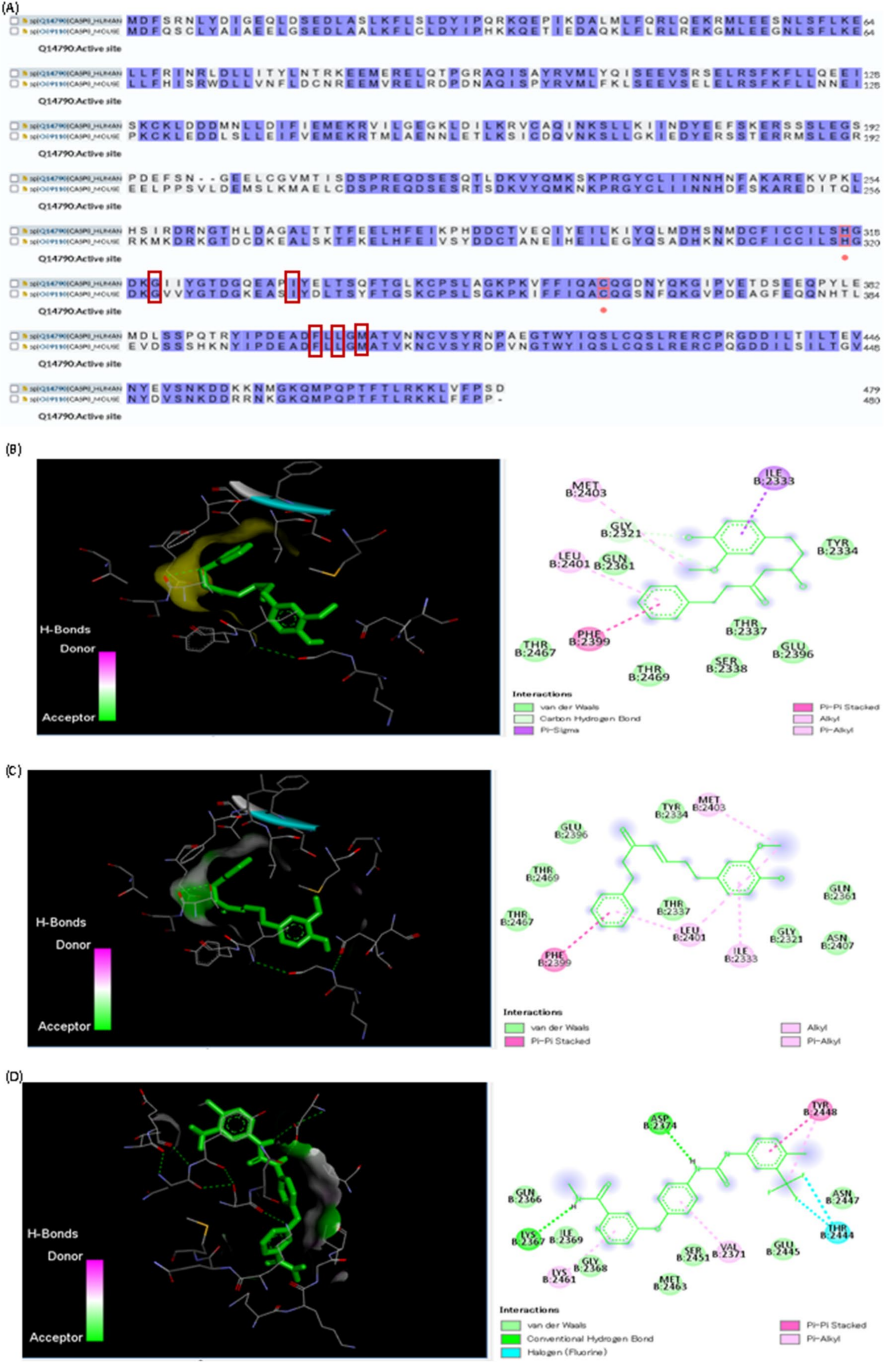
Sequence alignment and molecular docking. **A** Sequence alignment of human *CASP8 *(UniProt ID: Q14790) and mice caspase 8 (UniProt ID: O89110) was done through the UniProt database (https://www.uniprot.org/). **B** Molecular interaction of 5‐hydroxy‐7‐(4‐hydroxy‐3‐methoxyphenyl)‐1‐phenylheptan‐3‐one and human *CASP8 *(RCSB PDB ID: 1I4E). **C** Molecular interaction of 7‐(4″‐hydroxy‐3″‐methoxyphenyl)‐1‐phenylhept‐4‐en‐3‐one and human *CASP8 *(RCSB PDB ID: 1I4E). Red rectangular refers to binding sites for ligands. Red dots refer to active site residues

**Fig. 7:**
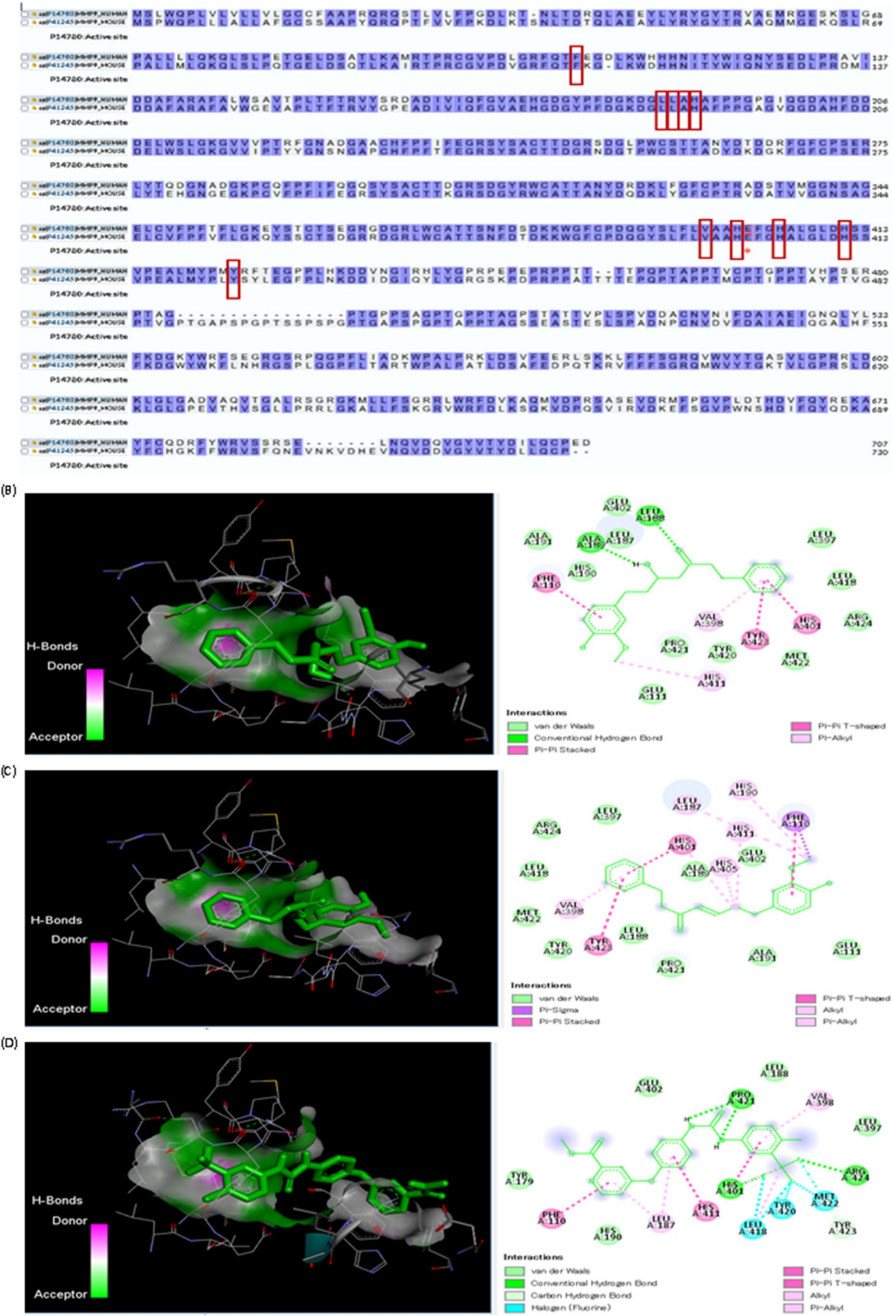
Sequence alignment and molecular docking. **A** Sequence alignment of human matrix metalloproteinase-9 (*MMP9*) (UniProt ID: P14780) and mice *CASP8* (UniProt ID: P41245) done through UniProt database (https://www.uniprot.org/). **B** Molecular interaction of 5‐hydroxy‐7‐(4‐hydroxy‐3‐methoxyphenyl)‐1‐phenylheptan‐3‐one and human *MMP9* (RCSB PDB ID: 1GKC). **C** Molecular interaction of 7‐(4″‐hydroxy‐3″‐methoxyphenyl)‐1‐phenylhept‐4‐en‐3‐one and human *MMP9* (RCSB PDB ID: 1GKC). The red rectangular area refers to the binding sites for ligands. Red dots refer to active site residue

**Fig. 8:**
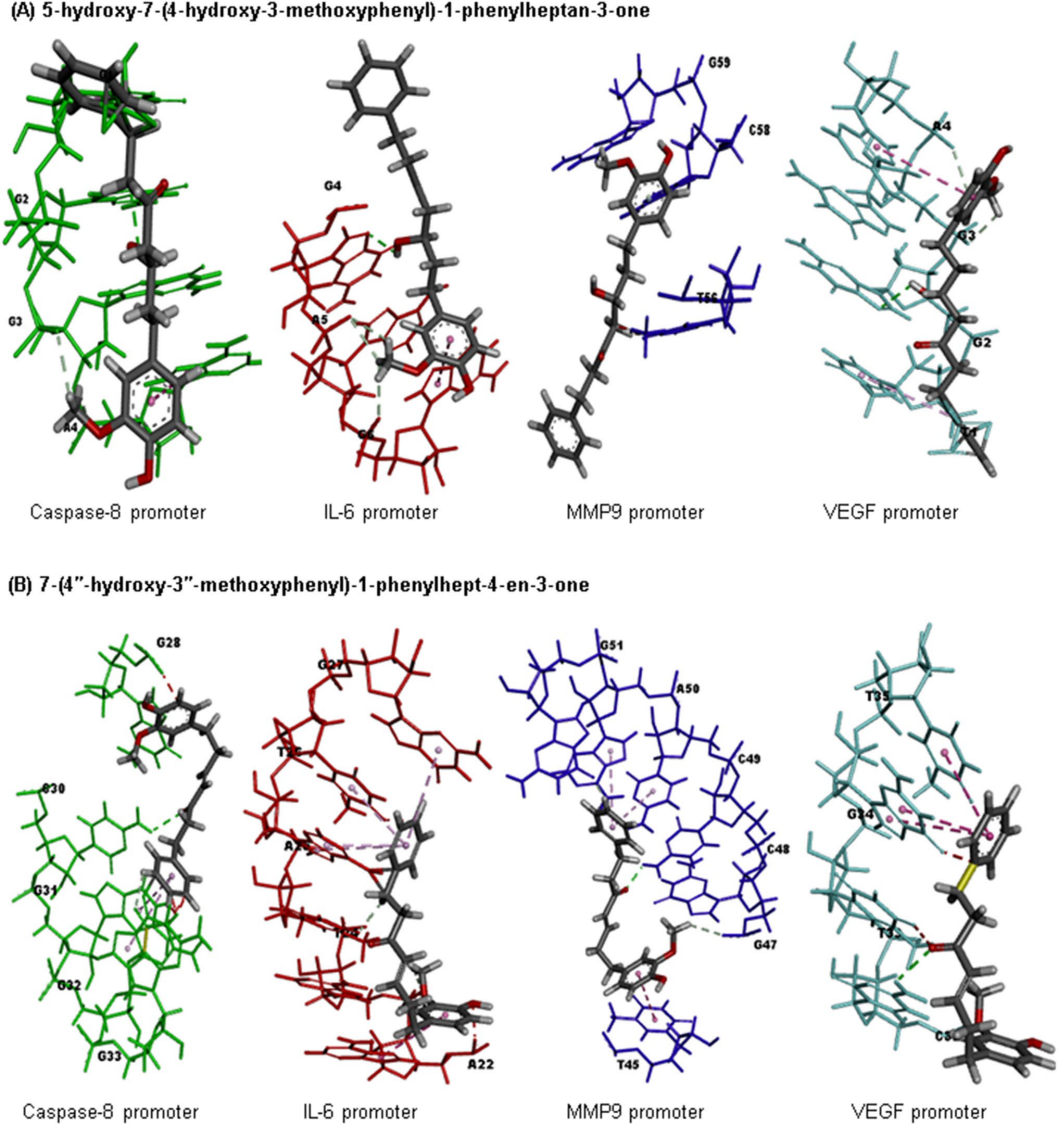
Molecular docking. **A** Molecular interaction of 5‐hydroxy‐7‐(4‐hydroxy‐3‐methoxyphenyl)‐1‐phenylheptan‐3‐one and mice *CASP8*, *IL-6*, *p53*, *MMP9*, and *VEGF* promotor genes. **B** Molecular interaction of 7‐(4″‐hydroxy‐3″‐methoxyphenyl)‐1‐phenylhept‐4‐en‐3‐one and mice *CASP8*, *IL-6*, *p53*, *MMP9*, and *VEGF* promotor genes

Molecular docking scores and interaction of DAH and SOR against *CASP8* were quantified in Table 2 and illustrated in Fig. 6. SOR exhibited the highest binding affinity (p*Ki=* 5.94), while 5‐hydroxy‐7‐(4‐hydroxy‐3‐methoxyphenyl)‐1‐phenylheptan‐3‐one and 7‐(4″‐hydroxy‐3″methoxyphenyl)‐1‐phenylhept‐4‐en‐3‐one were 4.62 and 4.77, respectively. The p*Ki* of 5‐hydroxy‐7‐(4‐hydroxy‐3‐methoxyphenyl)‐1‐phenylheptan‐3‐one (5.57), 7‐(4″‐hydroxy‐3″‐methoxyphenyl)‐1‐phenylhept‐4‐en‐3‐one (5.21), and SOR (7.19) are itemized in Table 2 and Fig. 7. Furthermore, DAH binds to the promotor sequences of *CASP8*, *IL-6*, *p53*, *MMP9*, and *VEGF* were revealed in Fig. 8.

**Table 2:** Docking score and interactions of DAH and SOR against human *CASP8* (target site PDB ID: 1I4E) and human *MMP9* (target site PDB ID: 1GKC)

Ligands	Caspase 8 (target site PDB ID: 1I4E)			MMP9 (target site PDB ID: 1GKC)		
	Binding free energy (kcal/mol)	p*Ki*	Ligand efficiency (kcal/mol/non-H atom)	Binding free energy (kcal/mol)	p*Ki*	Ligand efficiency (kcal/mol/non-H atom)
5‐Hydroxy‐7‐(4‐hydroxy‐3‐methoxyphenyl) ‐1‐phenylheptan‐3‐one	−6.3	4.62	0.2625	−7.6	5.57	0.3167
‐(4″‐Hydroxy‐3″‐methoxyphenyl) ‐1‐phenylhept‐4‐en‐3‐one	−6.5	4.77	0.2826	−7.1	5.21	0.3087
Sorafenib (SOR)	−8.1	5.94	0.2531	−9.8	7.19	0.3063

## Discussion

During this study, we sought to elucidate the role of DAH and/or sorafenib in ameliorating the anticancer efficacy of experimentally induced HCC by assessing the mechanism of action. In our study, DAH combined with SOR significantly enhanced its antiproliferative and cytotoxic effects on HepG2 cancer cells. HepG2 cells responded more positively to DAH and/or SOR since both compounds had lower IC_50_ values than WI-38 cells. This suggested that the combination of DAH and SOR was found to be more effective against HepG2 cells than either compound alone, suggesting that when used together, both compounds act synergistically in suppressing tumour growth. This further supports the notion that SOR is an effective treatment for hepatocellular carcinomas. After treating HepG2 cells with DAH at different doses, the IC_50_ values for SOR were significantly reduced. Our data showed that DAH showed the potential to act synergistically with SOR, thereby reducing the required dose. This could lead to a reduction of its side effects and improved efficacy, which would make SOR a safer and more effective chemotherapeutic drug for the potential treatment of liver cancer (Abdu et al. 2022).

Our data revealed that there was a significant increase in the levels of serum ALT, AST, and GGT in HCC mice, which could be explained by compromised cell membranes in certain types of hepatocytes. In line with current research findings, Huang et al. (2012), Badawy et al. (2021), and Abdu et al. (2022) have all reported similar findings. Accordingly, it was determined by Elzoghby et al. (2013) that both synthetic and natural anticancer medicines have the potential for antitumor activity when they have low liver damage enzyme levels. Furthermore, we found that treatment with DAH and/or SOR significantly reduced serum levels of these enzymes in HCC mice. The combination of DAH and/or SOR was the most effective treatment for reducing these enzymes. It is noteworthy that our results were in line with those of another study in which DAH was given to diabetic rats with type II diabetes to prevent hepatotoxicity (Heidari et al. 2022).

The primary gold standard for the detection of HCC is the AFP (Abdallah et al. 2022). In terms of AFP levels, the treatment with SOR and DAH was significantly associated with a decrease in levels, whether both treatments were administered alone or in combination with the highest level in HCC + SOR and HCC + DAH, respectively. Several studies have shown that the *A. officinarum* rhizome extract and its components are anticancer agents and that they possess potential anticancer activities against many cancer cell lines, such as the breast, neuroblastoma, liver, and lung (Basri et al. 2017).

In our study, it has been shown that DAH and/or SOR treatment decreased MDA and elevated T-SOD levels in HCC-bearing mice. The results of our research suggest that treatment with DAH and/or SOR significantly reduced oxidative stress levels. DAH, in accordance with this, stimulated T-SOD and decreased MDA levels in HepG2 cells, which supports this statement by enhancing free radical scavenging activity. As reported by Akazawa et al. (2006), the medicinal properties of this plant are mainly due to its DAH, which has diverse biological activities, including free radical scavenging, necessary for its therapeutic effects on cancer cells.

The docking results of our study demonstrated that DAH and SOR could bind to the active sites of *CASP8* and *MMP9* (Augoff et al. 2022), and the binding scores indicated that these compounds had a high affinity for the enzymes. Furthermore, the binding of the compounds to the promotor sequences of *CASP8*, *IL-6*, *p53*, *MMP9*, and *VEGF* suggested that the compounds could downregulate the expression of these genes. These findings were further supported by the biological analysis in our results, which exhibited that DAH and SOR could inhibit the activity of *CASP8* and *MMP9*.

Apoptosis is triggered by numerous stressors like DNA damage and oxidative stress, as well as *p53*, which activates the death receptor cascade or mitochondria-mediated cell damage (Zhivotovsky and Orrenius 2010). This expression was significantly elevated after DAH and/or SOR therapy, either separately or together. The expression of the *p53* gene may be triggered by the production of various DNA adducts, which too affect DNA replication, DNA helix distortion, and gene mutations. As a result of DAH treatment, HCC mice had higher *p53* expression (Mandlik and Mandlik 2021).

The expression of caspases mediates apoptosis, which is caused by the cleavage of hundreds of different proteins by caspases (Lopez and a. TS, , 2015). The relevant data confirmed that *CASP8* expressions were downregulated in the HCC group as compared to the control group. HCC + SOR + DAH had a dominant apoptotic impact compared to the HCC + SOR group, followed by the HCC + DAH group, and the HCC + DAH group had the lowest expression compared to the HCC untreated group. As a consequence of treatment with DAH and/or SOR, either in combination or individually, these expression levels significantly increased.

Some studies indicate that MMPs are a crucial component of regulating cell proliferation, invasion, and migration (Stetler-Stevenson and Anita 2001). In both cases, the expression of the *MMP9* protein was significantly upregulated by treatment with DAH and SOR, either individually or in combination. Kim et al. (2006) reported that a DAH from the medicinal plant *Alnus hirsuta* dissuades the transcription of *COX-2* and *MMP9* when applied to cultured human mammary epithelial cells.

As a result of the treatment with either DAH or SOR alone or in combination, the expression of *VEGF* was significantly increased. Ryan et al. (1998) reported that *VEGF* is one of the most potent and definitive angiogenesis regulators that are crucial for solid tumour growth. According to Hu et al. (2019), a diarylheptanoid blockade *VEGFR*-2-mediated signalling cascades in human umbilical vein endothelial cells (HUVECs). This information may offer new insights into the possibility of a DAH as an anti-angiogenesis therapeutic agent.

*IL-6* molecules are released from T cells and can induce B cell proliferation, differentiation, and antibody production (Liu et al. 2010). The expression of *IL-6* was significantly downregulated following treatment with DAH and SOR either alone or in combination, with the highest expression in the HCC + SOR + DAH group followed by the HCC + SOR group and lowest expression in HCC + DAH group, as compared with the HCC untreated group. DAH may function as an anti-inflammatory drug by specifically inhibiting *COX-2*, the synthesis of cytokines involved in the signalling cascade of inflammation, or the modification of pro-inflammatory gene expression (Vanucci-Bacqué and Bedos-Belval 2021).

**Fig. 9:**
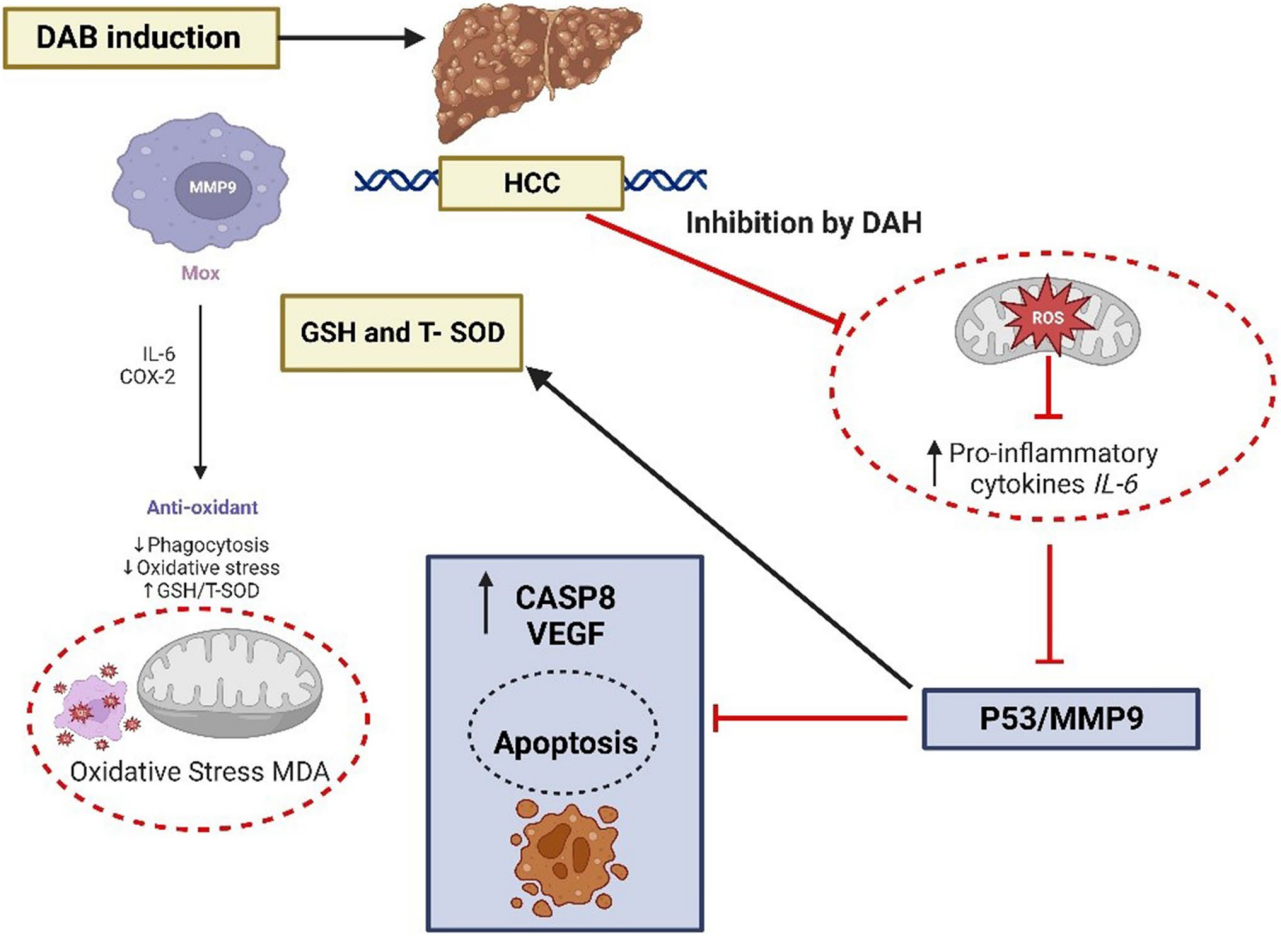
A schematic diagram illustrates DAH’s potential mode of action in vivo. With DAH treatment, *p53* and *MMP9* levels were downregulated as well as GSH and T-SOD levels were increased in the DAB-induced HCC

Therefore, these results supported our theory that DAH has an antitumor effect through targeting of *p53* and *MMP9* axis and that effect is critical to DAH-mediated HCC amelioration. Based on the study of HepG2 cells and *in vivo* studies, Fig. 9 summarizes the proposed antiproliferative and apoptotic action of DAH, as well as the downregulation of *p53*,* IL-6*,* CASP8*,* MMP9*, and *VEGF* genes *in vivo* by DAH as compared to control. The treated liver cancer cell line and hepatic genes were simulated to induce intrinsic and extrinsic pathways for apoptosis.

Regarding the histopathological examination, our findings also revealed a focal area of necrosis surrounded by hepatocytes with acidophilic cytoplasm and hyperchromatic nuclei, and a congested portal vein, along with loss of hepatic architecture with the presence of a focal area of hepatocellular degeneration with pyknotic nuclei and acidophilic cytoplasm as well as vacuolar degeneration of hepatocytes surrounded by inflammatory cells in the livers of patients with HCC. By treatment with DAH and/or SOR, the liver structure of HCC-bearing mice dramatically improved, particularly among those that were also treated with DAH and SOR. These results are consistent with those of Pathak and Khuda-Bukhsh (2007) who demonstrated that DAH is capable of producing substances that can inflict cellular injuries on cells of the liver as well as other tissues.

## Conclusions

In conclusion, DAH enhanced the cytotoxic and anti-tumour effects of SOR on HCC in the current study. This was likely due to the increased solubility of SOR in the presence of DAH, allowing for better absorption by tumour cells. As a result, the combination of the two drugs may have increased their cytotoxic effects, resulting in enhanced efficiency against cancer cells. Furthermore, administration of DAH and/or SOR to mice bearing HCC mitigated lipid peroxidation and tumour incidence. In addition, it increased antioxidant capacity and reversed the damage that had been caused to hepatocytes by HCC. In HCC-bearing mice, DAH and SOR are more effective at reducing the effects of liver injury. Moreover, our in vivo findings were confirmed and validated by the molecular docking study. This may therefore allow the application of this treatment as an adjunct to SOR as a cancer treatment method. A more in-depth study is therefore needed to implement a novel cancer-targeting strategy that incorporates DAH and SOR in co-therapy.

## Data Availability

The data presented in this study are available on request from the corresponding author.
